# Psychometric Assessment of the Need Satisfaction and Frustration Scale with Professional Romanian Athletes

**DOI:** 10.3390/ijerph19031696

**Published:** 2022-02-01

**Authors:** Dan Iulian Alexe, Beatrice Aurelia Abalasei, Gabriel Mares, Bogdan Constantin Rata, Teodora Mihaela Iconomescu, Georgeta Mitrache, Rafael Burgueño

**Affiliations:** 1 Department of Physical and Occupational Therapy, Faculty of Movement, Sports and Health, Sciences, “Vasile Alecsandri” University of Bacau, 600115 Bacau, Romania; alexedaniulian@ub.ro; 2 Department of Physical Education, Sports Faculty of Physical Education and Sports, “Al. I. Cuza” University of Iași, 700554 Iași, Romania; beatrice.abalasei@uaic.ro; 3 Sport Games and Physical Education Department, Faculty of Physical Education and Sports, “Dunărea de Jos” University of Galati, 800008 Galati, Romania; ticonomescu@ugal.ro; 4 Department of Teaching Staff Training, National University of Physical Education and Sports, 060057 Bucharest, Romania; georgeta.mitrache@unefs.ro; 5 Health Research Center, University of Almeria, Carretera de San Urbano, s/n, 04120 Almería, Spain; rmburgueno@ual.es

**Keywords:** basic psychological needs, autonomy, competence, relatedness, need satisfaction, need frustration, sportspeople

## Abstract

Background: Although athletes’ experiences of autonomy, competence, and relatedness play in key role in their motivation, performance-related outcomes, and wellness, there is no evidence to date on measures of autonomy, competence, and relatedness in the Romanian sport context. Building upon self-determination theory, the objective of this research was to adapt the Need Satisfaction and Frustration Scale and analyze its psychometric properties in the Romanian sport context. Methods: The participants were 642 professional athletes (354 males and 288 females; M_age_ = 22.81, SD = 5.78) who competed at the international and/or national level. Results: The results from confirmatory factor analyses psychometrically supported a six-factor correlated model, which was invariant across gender, age, and sport. Convergent validity was met by average variance extracted values between 0.60 and 0.74. Discriminant validity was underpinned by values from −0.72 to 0.72 for a heterotrait–monotrait ratio of correlations among the six factors. Reliability was endorsed by Cronbach’s alpha scores between 0.75 and 0.89, and between 0.76 and 0.89 for Raykov’s composite reliability coefficient. Criterion validity was supported by positive relationships of autonomy, competence, and relatedness satisfaction to autonomous motivation, and positive associations of autonomy, competence, and relatedness frustration with controlled motivation and amotivation. Conclusions: The Need Satisfaction and Frustration Scale is shown to be a valid and reliable measure of need satisfaction and frustration in professional Romanian athletes.

## 1. Introduction

A substantial body of research shows that athletes’ experiences of autonomy, competence, and relatedness perform an essential role in their motivation, performance-related outcomes, and wellness [1,2,3]. Self-Determination Theory (SDT) [4], particularly through the concept of Basic Psychological Needs (BPN), represents a broader theoretical framework that could provide a solid explanatory system for the “bright” and “dark” side of sport participation. While BPN satisfaction has been linked to autonomous motivation and adaptive consequences, contributing to better insight into the bright side of sport participation, BPN frustration has been associated with amotivation and maladaptive outcomes, providing a deeper understanding of the dark side.

Despite the importance of the distinction between need satisfaction and need frustration in explaining functioning patterns derived from sport participation, no instruments were found to measure athletes’ perceptions of need satisfaction and need frustration in the Romanian sport context. Hence, we sought to adapt the Need Satisfaction and Frustration Scale (NSFS) [5] to the sport context, and to gather validity and reliability evidence with professional Romanian athletes.

### 1.1. Basic Psychological Needs

A central tenet of SDT is the existence of the BPNs for autonomy, competence, and relatedness, considered to be universal and inherent nutrients for an individual’s growth, optimal functioning, and wellness [4]. SDT holds the premise that satisfaction BPN for autonomy (i.e., experiences of self-direction and personal endorsement in behaviors undertaken), competence (i.e., experiences of mastery and effectivity in achieving the desired goals), and relatedness (i.e., experiences of warmth, mutual care, and respect from significant others) energizes both intrinsic motivation and the optimal behavioral internalization, and contributes to proactivity, integrity, and wellness [6,7]. Conversely, SDT posits that BPN frustration—conceptualized as the active obstruction of the three BPNs [8]—hampers the internalization process and contributes to passivity, fragmentation, and illness [6,7]. When BPNs are being actively thwarted, autonomy frustration would be reflected by perceptions of being controlled by external or self-imposed pressures. Competence frustration would be expressed by perceived ineffectiveness and failure to attain accomplishment-related goals. Relatedness frustration would be manifested by perceived loneliness and social rejection [8].

Although at first sight need satisfaction and need frustration might theoretically fall into a single need fulfillment continuum as opposite ends [9], previous SDT-based studies have well documented that need satisfaction and need frustration constitute differentiated but related variables [10,11,12,13]. In particular, prior research argues that low levels of need satisfaction may simply mean need dissatisfaction in the sense that they did not suitably capture the active intensity and nature of feelings characterized by experiences of need frustration [12,14]. Need dissatisfaction is, therefore, thought to be the opposite end of need satisfaction in the need fulfillment continuum [15]. More specifically, need dissatisfaction would indicate the lack of any perception of positive characteristics defining a psychological experience (e.g., feelings of endorsement, acceptation, or understanding), but it fails to capture the presence of negative experimental characteristics (i.e., feelings of obligation, inefficacy, or rejection) [8]. To illustrate this, a person who experiences a low level of need satisfaction may indicate feelings of not having as many opportunities for choice as (s)he would like, not being very good at a specific task, and not being supported by significant others. Instead, an individual who perceives need frustration would express feelings of being forced into tasks, incompetent toward such activity, and excluded from a group. This distinction between need satisfaction and need frustration has postulated the existence of a bright side toward personal growth and wellness, and a dark side toward maladaptive functioning and illness [4,16].

SDT establishes that need satisfaction and need frustration may occur simultaneously in a specific context, with both distinctly contributing to predicting particular consequences [16]. In the sport context, a meaningful basis of research has analyzed the simultaneous effects of athletes’ perceptions of need satisfaction and need frustration on their behavioral regulation. These results displayed that, whereas need satisfaction was positively associated with autonomous motivation (i.e., the behavior is undertaken by experiences based on self-endorsement, psychological freedom, and volition), need frustration was positively related to controlled motivation (i.e., the behavior is undertaken by experiences based on coercion and external and self-imposed pressure) or amotivation (i.e., the absence of competence to perform, or the absence of value or interest in the behavior) [17,18,19,20,21]. Specifically, SDT-based research argues that intrinsic motivation (i.e., the behavior is adopted by enjoyment, curiosity, and seeing new perspectives), integrated regulation (i.e., the behavior is adopted by being congruent with the individual’s identity system) and identified regulation (i.e., the behavior is adopted by the recognition of its benefits) are autonomous forms of motivation [22]. While introjected regulation (i.e., the behavior is adopted by self-imposed pressures to gain self-esteem or to avoid shame and guilt) and external regulation (i.e., the behavior is adopted to obtain rewards or to avoid punishments) are understood as controlled forms of motivation [22].

### 1.2. Measuring Basic Psychological Needs in Sport

In research to date, there are no studies that analyze the adaptive and maladaptive consequences of the BPN being supported or thwarted in the sport environment for Romanian athletes due to the absence of well-validated instruments. At the international level, the first SDT-based instruments separately assessed the athletes’ perception of need-based experiences with specific measures of need satisfaction (see Gillet et al. [23] and Ng et al. [24]) and another of need frustration (see Bartholomew et al. 2011) [8]. Recently, new instruments were created by including the development of separate items to assess experiences both of need satisfaction and need frustration, such as the Basic Psychological Need Satisfaction and Frustration Scale (BPNSFS) [25], the Balanced Measures of Psychological Needs Scale (BMPNS) [26], and the Need Satisfaction and Frustration Scale (NSFS) [5]. Although the BPNSFS has been identified as the most commonly used measure of need satisfaction and need frustration at the international level, previous research has, instead, reported that the NSFS obtained a better psychometric performance than the BPNSFS and BMPNS [5]. It is also noteworthy that the NSFS is a briefer measure than the BPNSFS, which could represent an advantage in judging the satisfaction and frustration of each BPN [27].

The NSFS was developed by Longo et al. (2016), initially to measure need satisfaction and need frustration in the work and education settings. The scale consists of 18 items grouped into three items per factor to judge the satisfaction and frustration of each BPN. Longo et al. (2016, 2018) provided evidence in support of a six-factor correlated model against alternative models with three factors (i.e., autonomy, competence, and relatedness) and method corrections, and cross-loading models. In addition, discriminant validity was supported by values as high as 0.76 for the heterotrait–monotrait ratio of correlations [11], while criterion validity was gathered by positive associations of need satisfaction with wellness indicators, and positive relationships between need frustration and illness outcomes [5,11]. Likewise, a good level of reliability was obtained for each of the six factors comprising the scale with omega values between 0.69 and 0.85 [11], and Cronbach’s alpha values between 0.70 and 0.82 [5].

### 1.3. The Present Research

**Figure 1:**
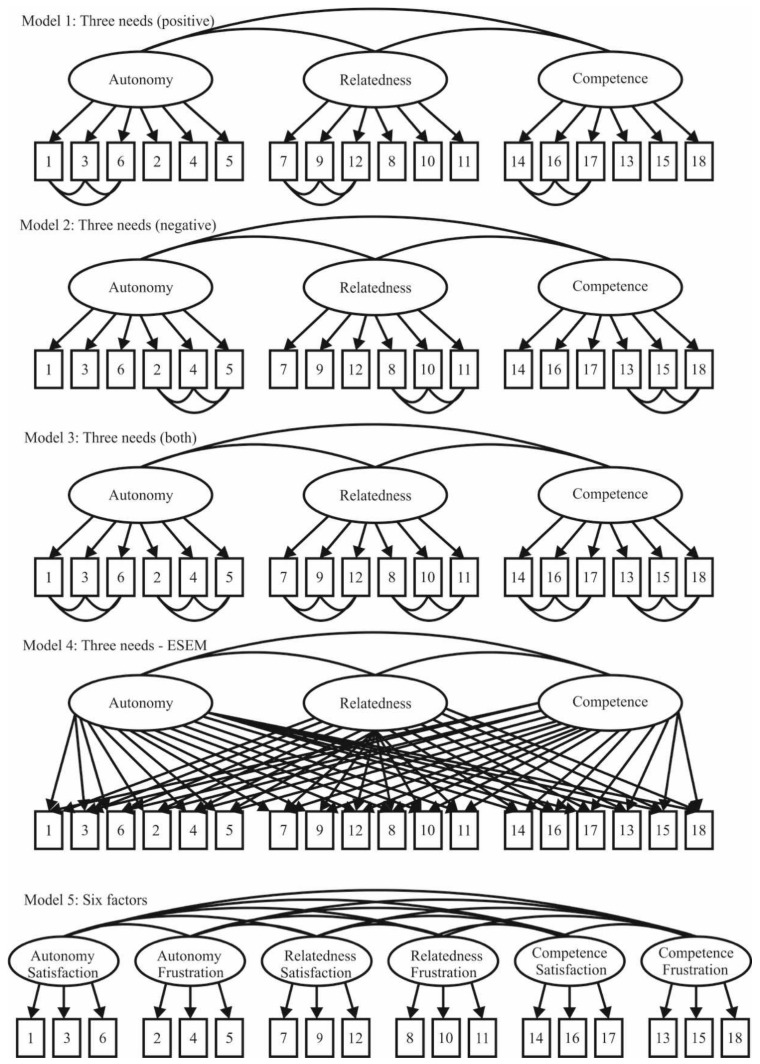
Hypothetical factor models.

Therefore, the objective of this research was to adapt the Need Satisfaction and Frustration Scale [5] to the Romanian sport context and to examine the psychometric properties of the resulting version with a sample of professional Romanian athletes. Consistent with previous studies [5,11,13], we tested the robustness of five factor models (see Figure 1): (a) three factors corresponding to the three BPNs, with correlated error terms among items from satisfaction of each BPN (Model 1); (b) three factors with correlated error terms between items from frustration of each BPN (Model 2); (c) three factors with correlated error terms among items from satisfaction and frustration of each BPN (Model 3); (d) a three-factor exploratory structural equation model (ESEM), in which all cross-loadings were calculated (Model 4); and (e) six-factor correlated model with satisfaction and frustration of each BPN as distinct but correlated factors (Model 5). Once the best-fit model was identified, we examined invariance across gender, age, and sport, in addition to running analyses of convergent validity, discriminant validity, and reliability. Lastly, we gathered criterion validity evidence by inspecting the predictive relationships of need satisfaction and need frustration on motivation. In accordance with research conducted on sport [17,18,19,20,21], we hypothesized that autonomy, competence, and relatedness satisfaction would positively predict autonomous motivation, while the frustration of each BPN would positively predict both controlled motivation and amotivation in professional athletes.

## 2. Materials and Methods

### 2.1. Design

This research took an instrumental design [28] because it aimed at examining the psychometric properties of a measurement instrument.

### 2.2. Participants

The participants were 642 athletes (354 sportsmen and 288 sportswomen) aged between 18 and 52 years old (M = 22.81, SD = 5.78). They were involved in both individual sports (n = 305), such as athletics, gymnastics, rowing, cycling, weightlifting, and swimming, and team sports (n = 337), such as soccer, basketball, rugby, handball, volleyball, ice hockey, and water polo. The athletes’ competitive experiences ranged from 7 to 28 years (M = 11.05, SD = 5.00) at the international or national level.

### 2.3. Instruments

#### 2.3.1. Basic Psychological Need Satisfaction and Frustration in Sport

We utilized the Romanian version adapted to sport of the Need Satisfaction and Frustration Scale (NSFS, see Table A1) [5] to judge athletes’ perceptions of BPN satisfaction and frustration in sport. The scale consists of six 3-item factors that measure autonomy satisfaction (e.g., “I feel I’m given a lot of freedom in deciding how I do things”), competence satisfaction (e.g., “I feel I am very good at the things I do”), relatedness satisfaction (e.g., “I feel the people I interact with really care about me”), autonomy frustration (e.g., “I feel forced to follow directions regarding what to do”), competence frustration (e.g., “I doubt whether I am able to carry out my tasks properly”), and relatedness frustration (e.g., “I feel alone when I’m with other people”). It is preceded by the sentence “In my training and competitions…” and items are scored on a 7-point Likert scale ranging from 1 (strongly disagree) to 7 (strongly agree).

#### 2.3.2. Motivation in Sport

We administrated the Romanian version (masked details for review) of the Behavioral Regulation in Sport Questionnaire [29] to assess athletes’ perceptions of motivation toward sport. The instrument includes six 4-item factors that measure intrinsic motivation (e.g., “Because I enjoy it”), integrated regulation (e.g., “Because it’s a part of who I am”), identified regulation (e.g., “Because I value the benefits of my sport”), introjected regulation (e.g., “Because I would feel ashamed if I quit”), external regulation (e.g., “Because I feel pressure from other people to play”), and amotivation (e.g., “But I question why I continue”). It is preceded by the sentence “I participate in my sport…” and items are scored on a 7-point Likert scale ranging from 1 (strongly disagree) to 7 (strongly agree). In line with SDT [7] and following prior research in sport [30], a mean score for autonomous motivation was computed by taking mean values of intrinsic motivation and integrated and identified regulation, while a mean score for controlled motivation was estimated by averaging mean values of introjected and external regulation.

### 2.4. Procedure

To adapt the NSFS to the Romanian sport domain, we followed the guidelines established by the International Test Commission [31]. First, a backward-translation strategy was used, in which a panel of two translators translated the instrument into Romanian, and then another panel of two translators translated it into English. Second, the equivalence between the two versions and the original version was qualitatively analyzed and verified by the main author of this manuscript. Third, a new panel of two experts in sport psychology adapted the content of each item to make it applicable to the sport setting. Fourth, another panel of two experts in sport psychology qualitatively assessed the items’ content to determine their suitability for measurement of the psychological variable intended. Fifth, a pilot study was developed with 11 athletes who completed the instrument and reported the correct understanding of the items’ content. Thus, this adaptation and translation process provided validity evidence based on the instrument’s content.

In order to recruit and select participants, we utilized a convenient sampling method according to previous research in sport [29]. Indeed, the potential participants had to comply with the following inclusion criteria: (a) older than 18 years old, and (b) professional athlete who competed at the international and/or Romanian national level. We contacted different Romanian sports federations and professional sports clubs to request collaboration and inform them of the research’ objectives. After obtaining their approval to proceed with data collection, 800 athletes were invited via e-mail to participate in this research. A total of 650 (81.25%) athletes responded, agreeing to voluntary participation in this research. Once the informed written consent from all the participating athletes was obtained, we sent them an online questionnaire survey via email. To fill it in, we provided information regarding voluntary and anonymous participation, as well as the absence of right and false responses given that we only aimed to know their perceptions of their sport. The approximate mean time was 20 min. We obtained the ethical approval from the board of the of the “Vasile Alecsandri” University of Bacau (code: 21504/2/17.2.2021).

### 2.5. Data Analysis

Statistical data analyses were conducted using the Statistical Package for the Social Sciences (IBM SPSS Statistics for Windows, version 25.0; Armonk, NY, USA) and the extended version for the analysis of structural equation modelling AMOS (version 25.0; Armonk, NY, USA). Before the main analyses, the dataset was screened to detect potential univariate and multivariate outliers. Particularly, three univariate outlier (i.e., Z < 3) and five multivariate outliers (i.e., Mahalanobis distance at *p* < 0.001) were identified. They were removed from the subsequent analyses, and the final sample included 642 professional athletes.

The five factor models (see Figure 1) were tested using the maximum likelihood method accompanied by the bootstrapping technique with 5000 iterations given the absence of multivariate normality (Mardia’s coefficient = 122.50, *p* < 0.01) [32]. To judge the goodness of fit for every model, a combination of fit indexes was used: ratio between χ^2^ and degrees of freedom (χ^2^/*df*), Comparative Fit Index (CFI), Tucker-Lewis Index (TLI), Standardized Root Mean Square Residual (SRMR), Root mean Square Error of Approximation (RMSEA), accompanied by its confidence internal at 90% (90% CI) and *p*-close, and Akaike Information Criterion (AIC). Values up to 5 for the χ^2^/*df* ratio, above 0.95 for CFI and TLI, and below 0.060 for SRMR and RMSEA are considered as representative of a good degree of fit to data [33]. AIC is commonly used to select the best-fit model among competing models, indicating that the model with the smallest value would be the one selected [32]. Standardized residual covariances are acceptable with absolute values less than 2.58, while standardized regression weights are suitable with values over 0.50 [34].

In accordance with Putnick and Bornstein (2016), three multi-group analyses were performed to examine the tenability of four types of invariance: configural invariance (no equality constrains, Model 1), weak invariance (equal item factor loadings, Model 2), strong invariance (equal item factor loadings and item intercepts concurrently, Model 3), and strict (equal item factor loadings, item intercepts, and item residuals concurrently, Model 4). To determinate invariance in multi-group analyses, differences as high as 0.010 in CFI values and up to 0.015 in RMSEA values among two successively constrained models are considered to be indicative of the instrument’s invariance [35]. To test age invariance, a median was estimated to create two age groups. The first group (i.e., younger athletes) consisted of 338 athletes aged between 18 and 21 years old (M = 19.54, SD = 1.64), while the second group (i.e., older athletes) included 304 athletes aged between 22 and 52 years old (M = 27.55, SD = 4.98).

Convergent validity evidence was gathered by estimating average variance extracted (AVE), displaying that values higher than 0.50 are acceptable [34]. Discriminant validity evidence was met via correlations among latent variables, heterotrait–monotrait (HTMT) ratio of correlations [36], and the comparison of AVE with maximum shared variance (MSV) and average shared variance (ASV). Requirements for discriminant validity are met by values less than 0.85 for correlations and HTMT [32,36] and AVE values higher than MSV and ASV values, respectively [34]. Moreover, reliability evidence was given through Cronbach’s alpha and Raykov’s compositive reliability coefficient. Both are appropriate with values over 0.70 [37].

Criterion validity evidence was examined through three linear regression analyses. In each analysis, autonomy, competence, and relatedness satisfaction and frustration were introduced as independent variables, while autonomous motivation, controlled motivation, and amotivation were, respectively, considered as dependent variables. Lastly, descriptive statistics (i.e., mean scores and standard deviation) were reported for each variable under study.

## 3. Results

### 3.1. Confirmatory Factor Analysis

Goodness-of-fit measures obtained in the different tested models are shown in Table 1. The only model that obtained a good fit to the observed data was the six-factor correlated model. Indeed, this factor model displayed the smallest AIC value, making it the most parsimonious and, therefore, the best-fit model in comparison with the other tested models. In this way, the six-factor correlated model was the one chosen for the remaining analyses.

**Figure 2:**
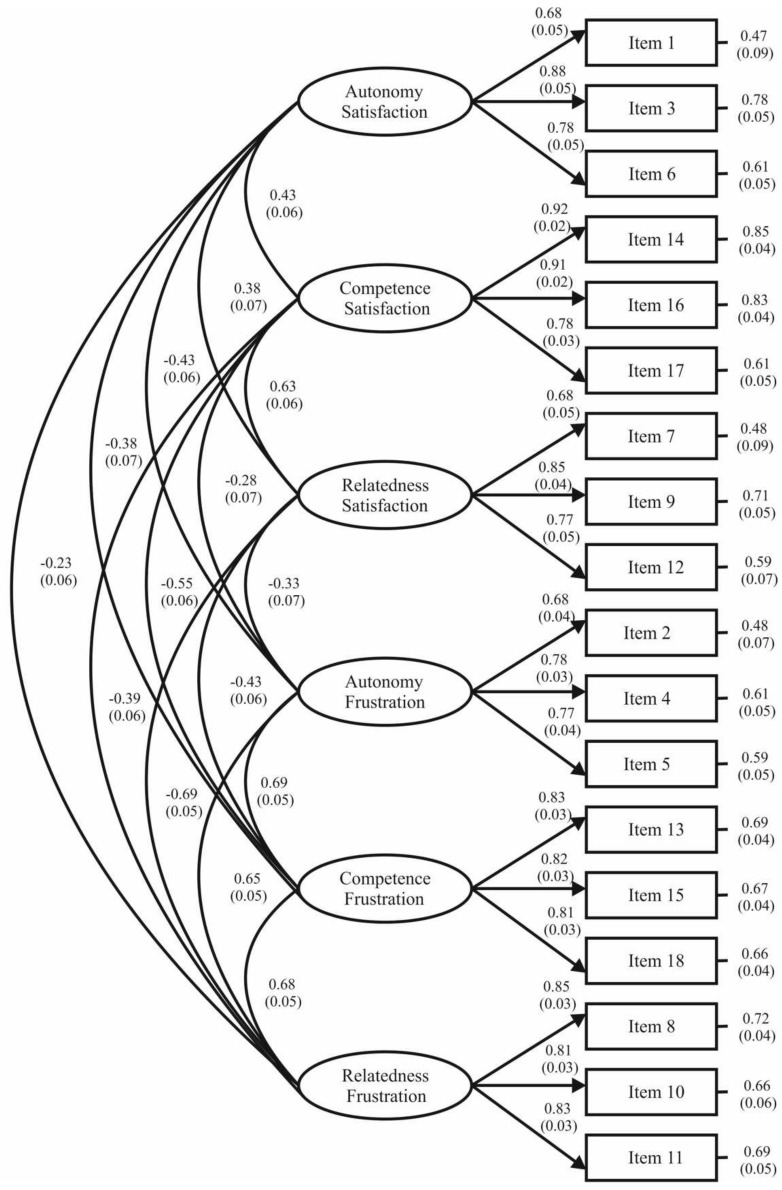
Confirmatory factor analysis of the Romanian sport version of the Need Satisfaction and Frustration Scale.

Regarding the six-factor correlated model, Figure 2 shows standardized regression weights ranging from 0.71 to 0.88, with each being statistically significant (*p* < 0.001). Correlations between latent factors oscillated between −0.69 and 0.69. Specifically, positive correlations were found among the satisfaction of the three BPN, in the same way that autonomy, competence, and relatedness frustration were positively correlated. There were also negative correlations between the satisfaction and the frustration of the three BPN. Lastly, the analysis of standardized residual covariances revealed values between −2.35 and 2.37.

### 3.2. Multi-Group Analysis of Invariance

Results of the three multi-group analyses of invariance are shown in Table 2. Particularly, there were differences lower than 0.010 in CFI values and less than 0.015 in RMSEA values between each two successively constrained models. Therefore, evidence was gathered to support invariance across gender, age, and sport for the instrument.

### 3.3. Analysis of Convergent and Discriminant Validity and Reliability

Results of the convergent validity analysis are presented in Table 3. The estimation of AVE revealed scores from 0.60 to 0.74. Likewise, Table 3 also shows the results of discriminant validity analysis. Specifically, there were AVE values higher than MSV and ASV values for each of the six factors, as well as correlations ranging from −0.69 to 0.69 and HTMT scores between −0.72 and 0.72. On the other hand, results emerging from reliability analysis displayed suitable scores for each one of the six factors, with Cronbach’s alpha values between 0.75 and 0.89, and Raykov’s coefficients between 0.76 and 0.89.

### 3.4. Linear Regression Analyses

Results from the three linear regression analyses are shown in Table 4. In particular, autonomy, competence, and relatedness satisfaction positively predicted autonomous motivation (β = 0.13, *p* = 0.002; β = 0.37, *p* < 0.001; β = 0.16, *p* < 0.001), while controlled motivation and amotivation were positively predicted by frustration of the need for autonomy (β = 0.14, *p* = 0.005; β = 0.15, *p* < 0.001), competence (β = 0.16, *p* = 0.006; β = 0.28, *p* < 0.001), and relatedness (β = 0.19, *p* < 0.001; β = 0.14, *p* = 0.008). In addition, competence satisfaction negatively predicted amotivation (β = −0.11, *p* = 0.020). The total variance explained accounted for 29% in autonomous motivation, 18% in controlled motivation, and 33% in amotivation.

### 3.5. Descriptive Statistics

Descriptive statistics for the satisfaction and frustration of every BPN and the three types of motivation are presented in Table 5. In particular, mean scores for autonomy, competence, and relatedness satisfaction and autonomous motivation were higher than the scale midpoint. Instead, mean values were lower than the midpoint of the measurement scale for autonomy, competence and relatedness frustration, controlled motivation and amotivation.

## 4. Discussion

The objective of this research was to adapt and to gather validity and reliability evidence of the Need Satisfaction and Frustration Scale [5] in the Romanian sport context. Overall, the results obtained in this research gather validity and reliability evidence in support of the NSFS as a psychometrically robust measure of the professional athletes’ perception of need satisfaction and need frustration in the Romanian realm.

Consistent both with the original version [5] and the Spanish version [11] of the instrument, the results from the different confirmatory factor analyses showed that the six-factor correlated model obtained the best fit. These findings provided evidence to underpin the premise that need satisfaction and need frustration are two separate variables in line with the SDT assumptions [4]. Moreover, the analysis of standardized residual covariances showed absolute values lower than 2.58, which permitted us to exclude the presence of misspecifications in the instrument’s factor structure and verify, thereby, the absence of discrepancies between the hypothesized model tested and the observed data. Furthermore, the examination of standardized regression weights displayed scores above 0.50, with each being significant, reflecting that each of the 18 items adequately represented the target theoretical factor to be measured.

The findings that emerged from the three multi-group analyses underpinned the instrument’s invariant character by suggesting that factor loadings (weak invariance), intercepts (strong invariance), and error variances (strict invariance) are equivalent across gender, age, and sport. Unlike previous research conducted on this instrument [5,11], this is, indeed, the first study that provided evidence in support of invariance for the NSFS, which provides new validity evidence based on the internal structure for this scale. In practice, the examination of invariance is an important point given that it allows us to compare and inspect the possible differences in the professional athletes’ perception of need satisfaction and frustration in the sport context between sportsmen and sportswomen with different ages and type of sport practiced.

On the other hand, the first convergent validity evidence for the NSFS was provided in this study with AVE scores over 0.50 for each one of the six factors comprising it, implying that items from each factor appropriately captured the meaning of such factor. Regarding the instrument’s discriminant validity, the results of this study were similar to the ones obtained in Longo et al.’s study [11], with HTMT values lower than 0.85, indicating that the six separate factors are differentiated constructs. Indeed, both values between −0.69 and 0.60 in correlations, and AVE values higher than MSV and ASV values for each of the six factors provided new evidence in support of discriminant validity among factors, as well as strengthened the good level of conceptual discrimination between latent variables. In line with previous studies [5,11], suitable values were obtained for Cronbach’s alpha and Raykov’s composite reliability coefficient, showing a good level of reliability for each of the six factors of the NSFS in the Romanian sport domain.

The results obtained in the structural equation modeling provided criterion validity evidence for the Romanian sport version of the NSFS. They are in line with the hypotheses proposed in this research and with previous research conducted in sport [17,18,19,20,21]. Particularly, need satisfaction positively predicted autonomous motivation, while need frustration positively predicted both controlled motivation and amotivation. These findings indicate how both professional athletes’ perceptions distinctly contributed to explaining specific motivational outcomes in the sport context, aligning with the tenets described by SDT [6,7]. Indeed, these findings demonstrated that need satisfaction plays an energizing role in developing intrinsic motivation and the optimal behavioral internalization (i.e., integrated and identified regulation). A plausible explanation would be that need satisfaction contributes to developing internal sources that ease dealing with those maladaptive environmental circumstances [16]. Furthermore, it is noteworthy that competence satisfaction also performs a buffering role against the athletes’ maladaptive experiences linked to amotivation in sport. In contrast, these results pointed out that need frustration directly leads to amotivation as well as a nonoptimal internalization, implying the behavior would be undertaken by external or self-imposed pressures. Thus, the results underscore that while need satisfaction yields a deeper insight into the bright side of motivation, need frustration provides a better understanding of the dark side of motivation.

### 4.1. Practical Implications

The validation of the NSFS to the Romanian sport context permits us to measure the professional athletes’ perception of need satisfaction and need frustration in Romania, which entails a series of implications. At the methodological level, the Romanian version of the NSFS represents the first well-validated instrument measuring need satisfaction and frustration perceived by Romanian athletes. At the theoretical level, our results strengthen the premise that need satisfaction and need frustration should be understood as two distinguishable but correlated variables, with each better predicting adaptive and maladaptive consequences, respectively. Likewise, and in line with SDT [6], these findings support the tenet of the existence both of a bright motivational pathway toward growth and wellness and a dark motivational pathway toward nonoptimal functioning patterns and illness. Furthermore, the assessment of need satisfaction and need frustration might help to gain a deeper understanding of the Romanian athletes’ need-based experiences involved in sport. At the practical level, the results open the way for developing potentially effective interventions by indicating that increased athletes’ levels of autonomy, competence, and relatedness need satisfaction may foster their autonomous motivation and optimal behavioral internalization. Furthermore, the Romanian sport version of the NSFS could be utilized to examine changes in athletes’ need-based experiences during a single season, and to analyze their trajectories in terms of need satisfaction and frustration throughout their sports careers. As a whole, this information will help coaches to adopt more adapted coaching strategies to develop autonomy, competence, and relatedness satisfaction and, in turn, to avoid experiences related to need frustration in professional athletes.

### 4.2. Limitations

Although this research has gathered evidence demonstrating the validity and reliability of the NSFS as a psychometrically robust measure of need satisfaction and frustration among Romanian professional athletes, a series of limitations need to be indicated. First, our results were based on a convenience sample, which does not allow us to generalize them to the population as a whole. Therefore, new studies are required to extend the validity and reliability of the NSFS in other life’s domains using individuals with different characteristics. Furthermore, there is a need to expand the instrument’s invariance with more heterogeneous samples of athletes regarding ethical background, religion, injuries, performance level, or sport experience in order to determine if the instrument equally performs across such groups. Second, the cross-sectional design makes it impossible to establish cause-effect relationships between the need satisfaction and frustration, and motivation. Therefore, longitudinal research is required to analyze the association of the perception of need satisfaction and frustration with the three types of motivation. Third, this research failed to consider the specific point of the season of the participating athletes, which could somehow influence the contextual measurement of the athletes’ need-based experiences in the sports settings. Thus, future research should take into account this aspect when tackling the analysis of need satisfaction and need frustration in professional athletes.

## 5. Conclusions

The results of the present study provide evidence in support of the Romanian sport version of the NSFS, indicating that it is a valid and reliable measure of need satisfaction and need frustration among professional athletes. It is expected that this instrument eases SDT-based research on the differentiated effects of need satisfaction and need frustration on the specific psychological experiences exhibited by professional athletes during their training and competitions.

## Figures and Tables

**Table 1:** Goodness-of-fit measures for the tested models.

	χ^2^ (*df*)	χ^2^/*df*	CFI	TLI	SRMR	RMSEA (90% CI; *p*-Close)	AIC
Three needs (positive)	974.742 (123)	7.925	0.882	0.854	0.080	0.104 (0.098–0.110; <0.001)	1070.742
Three needs (negative)	1433.744 (123)	11.656	0.819	0.775	0.120	0.129 (0.123–0.135; <0.001)	1529.744
Three needs (both)	570.947 (114)	5.008	0.937	0.915	0.064	0.079 (0.073–0.086; <0.001)	684.947
Three needs—ESEM	854.477 (102)	8.377	0.896	0.855	0.070	0.017 (0.101–0.114; <0.001)	992.477
Six factors	376.605 (120)	3.138	0.967	0.955	0.042	0.042 (0.036–0.048; 0.990)	564.605

**Table 2:** Multi-group analysis of invariance.

Invariance across Gender							
	χ^2^ (*df*)	CFI	RMSEA (90% CI)	MC	Δχ^2^ (Δdf)	ΔCFI	ΔRMSEA
1. Configural invariance	431.03 (240)	0.954	0.049 (0.043–0.055)	-	-	-	-
2. Weak invariance	450.89 (252)	0.952	0.049 (0.043–0.054)	1 vs. 2	19.86 (12) *	−0.002	0.000
3. Strict invariance	518.50 (270)	0.943	0.051 (0.046–0.056)	2 vs. 3	67.29 (18) ***	−0.009	0.002
4. Strong invariance	568.50 (288)	0.937	0.052 (0.046–0.057)	3 vs. 4	50.32 (18) ***	−0.006	0.001
Invariance across Age							
	χ^2^ (*df*)	CFI	RMSEA (90% CI)	MC	Δχ^2^(Δdf)	ΔCFI	ΔRMSEA
1. Configural invariance	376.61 (240)	0.967	0.042 (0.036–0.048)	-	-	-	-
2. Weak invariance	403.12 (252)	0.964	0.042 (0.037–0.048)	1 vs. 2	26.51 (12) *	−0.003	0.000
3. Strict invariance	467.33 (270)	0.956	0.045 (0.040–0.050)	2 vs. 3	65.21 (18) ***	−0.008	0.003
4. Strong invariance	520.60 (288)	0.950	0.046 (0.041–0.051)	3 vs. 4	53.27 (18) ***	−0.006	0.001
Invariance across Sport							
	χ^2^ (*df*)	CFI	RMSEA (90% CI)	MC	Δχ^2^ (Δdf)	ΔCFI	ΔRMSEA
1. Configural invariance	416.55 (240)	0.961	0.046 (0.040–0.051)	-	-	-	-
2. Weak invariance	430.76 (252)	0.960	0.045 (0.039–0.051)	1 vs. 2	14.21 (12)	−0.001	−0.001
3. Strict invariance	481.60 (270)	0.954	0.046 (0.041–0.051)	2 vs. 3	50.86 (18) ***	−0.006	0.001
4. Strong invariance	512.74 (288)	0.952	0.046 (0.040–0.051)	3 vs. 4	31.16 (18) *	−0.002	0.000

Note: MC = Models comparison, vs. = versus, *** *p* < 0.001, * *p* < 0.05.

**Table 3:** Reliability coefficients and convergent and discriminant validity.

	α	ρ	AVE	MSV	ASV	1	2	3	4	5	6
1. Autonomy satisfaction	0.78	0.78	0.64	0.26	0.21	-	0.43	0.38	−0.43	−0.38	−0.23
2. Competence satisfaction	0.89	0.89	0.74	0.48	0.29	0.51	-	0.63	−0.28	−0.55	−0.39
3. Relatedness satisfaction	0.75	0.76	0.61	0.53	0.34	0.53	0.72	-	−0.33	−0.43	−0.69
4. Autonomy frustration	0.81	0.82	0.60	0.47	0.29	−0.53	−0.36	−0.45	-	0.69	0.65
5. Competence frustration	0.87	0.87	0.68	0.55	0.37	−0.43	−0.66	−0.51	0.71	-	0.68
6. Relatedness frustration	0.87	0.87	0.69	0.55	0.38	−0.34	−0.47	−0.72	0.70	0.51	-

Note: Numbers above diagonal display correlations from confirmatory factor analysis, and numbers below diagonal show heterotrait–monotrait ratio of correlations.

**Table 4:** Predictive effects of need satisfaction and need frustration on the three types of motivation in professional athletes.

	Autonomous Motivation			Controlled Motivation			Amotivation		
	β	t	R^2^	β	t	R^2^	β	t	R^2^
(constant)	-	16.26	0.29	-	1.57	0.18	-	3.45	0.33
Autonomy Satisfaction	0.13 **	3.16		−0.05	−1.22		−0.03	−0.88	
Competence Satisfaction	0.37 ***	7.71		−0.02	−0.44		−0.11 *	−2.33	
Relatedness Satisfaction	0.16 ***	3.39		0.05	0.92		−0.05	−1.04	
Autonomy Frustration	−0.02	−0.43		0.14 **	2.83		0.15 ***	3.39	
Competence Frustration	−0.01	−0.10		0.16 **	2.76		0.28 ***	5.60	
Relatedness Frustration	−0.04	−0.70		0.23 ***	3.99		0.14 **	2.67	

Note: *** *p* < 0.001, ** *p* < 0.01, * *p* < 0.05.

**Table 5:** Descriptive statistics for each target variable.

	Range	M (SD)	Skewness	Kurtosis
Autonomy satisfaction	1–7	5.09 (1.57)	−0.47	−0.59
Competence satisfaction	1–7	5.55 (1.31)	−0.67	−0.28
Relatedness satisfaction	1–7	5.54 (1.37)	−0.71	−0.32
Autonomy frustration	1–7	3.21 (1.57)	0.23	−0.93
Competence frustration	1–7	2.99 (1.64)	0.48	−0.71
Relatedness frustration	1–7	2.79 (1.58)	0.56	−062
Autonomous motivation	1–7	6.23 (0.89)	−1.76	1.60
Controlled motivation	1–7	2.20 (1.30)	1.40	1.55
Amotivation	1–7	2.12 (1.49)	1.52	1.48

## Data Availability

Data is available upon request.

## Appendix A

**Table A1:** Romanian version of the Need Satisfaction and Frustration Scale in sport. In my training and competitions… [În timpul antrenamentelor și competițiilor mele…].

Autonomy satisfaction
1. I feel I’m given a lot of freedom in deciding how I do things [Simt că mi se acordă multă libertate în a decide cum să procedez]
3. I feel completely free to make my own decisions [Mă simt complet liber(ă) să iau propriile decizii]
6. I feel free to decide what to do [Mă simt liber să decid ce să fac]
**Autonomy frustration**
2. I feel I am prevented from choosing the way I carry out tasks [Mă simt împiedicat(ă) în a alege felul de executare a sarcinilor]
4. I feel forced to follow directions regarding what to do [Mă simt forțat(ă) să urmez anumite ordine cu referitoare la ce trebuie să fac]
5. I feel under pressure to follow standard procedures [Mă simt presat(ă) de a urma proceduri standard]
**Relatedness satisfaction**
7. I feel the people I interact with really care about me [Simt că oamenii cu care interacționez țin cu adevărat la mine]
9. I feel I’m perfectly integrated into a group [Mă simt perfect integrat în grup]
12. I feel very close and connected with other people [Mă simt foarte apropiat și conectat cu alți oameni]
**Relatedness frustration**
8. Sometimes, I feel a bit rejected by others [Uneori, mă simt puțin respins(ă) de alții]
10. I feel a bit alone when I’m with other people [Mă simt puțin singur(ă) atunci când sunt cu alți oameni]
11. On occasions, I feel people are a bit cold towards me [Câteodată simt că oamenii sunt puțin reci față de mine]
**Competence satisfaction**
14. I feel I am very good at the things I do [Simt că sunt foarte bun(ă) la ceea ce fac]
16. I feel highly effective at what I do [Mă simt foarte eficient în ceea ce fac]
17. I feel I can accomplish even the most difficult tasks [Simt că pot duce la bun sfârșit până și cele mai dificile sarcini].
**Competence frustration**
13. I doubt whether I am able to carry out my tasks properly [Am îndoieli că pot să îmi îndeplinesc sarcinile în mod corespunzător]
15. Occasionally, I feel incapable of succeeding in my tasks [Mă simt uneori incapabil să finalizez sarcinile mele]
18. I sometimes feel unable to master hard challenges [Uneori mă simt incapabil(ă) de a duce la bun sfârșit sarcinile dificile]

Note: Items from the Romanian version are shown into square brackets.
